# Supplementary material for: Selective carbon sources influence the end products of microbial nitrate respiration
Source: ISME J. 2020 May 5;14(8):2034–45. doi: 10.1038/s41396-020-0666-7 (PMC7368043; doi:10.1038/s41396-020-0666-7)
Supplement: Supplementary file 1 — Captions for Supplemental Figures [file 41396_2020_666_MOESM1_ESM.docx]

**Supplemental materials**

**Figure S1. A.**  Nitrite concentrations and donor electron equivalents for the Jewel Lake enrichment recovered on 10 different carbon sources in triplicate (Pearson’s *r* = -0.22, *p* = 0.1623). **B.** Nitrite concentrations and pH for the Jewel Lake enrichment recovered on 10 different carbon sources in triplicate (Pearson’s *r* = 0.3625, *p* = 0.052). **C.**  Ammonium concentrations and donor electron equivalents for the Jewel Lake enrichment recovered on 10 different carbon sources in triplicate (Pearson’s *r* = -0.2617, *p* = 0.1623). **D.** Ammonium concentrations and pH for the Jewel Lake enrichment recovered on 10 different carbon sources in triplicate (Pearson’s *r* = -0.11, *p* = 0.5591). Points are colored based on which dominant strain is most highly selectively enriched in each condition.

**Figure S2.** Ammonium (A-E) or nitrite concentrations in the Jewel Lake enrichment recovered on different carbon sources plotted against relative abundances of *Escherichia* and *Klebsiella* (A), *Citrobacter* (B), *Pseudomonas* and *Sulfurospirillum* (C), or *Clostridium* and *Peptostreptococcaceae* (D) strains*.*

**Dataset S1**

**Table S1.** Carbon source influence on the end-products of nitrate reduction in enrichment cultures.

**Table S2.** Taxonomy and genetic functional potential of strains in Jewel Lake enrichment culture

**Table S3.** Functional activity and community composition of Jewel Lake enrichment recovered on various carbon sources.

**Table S4.** MetaBat2 and CheckM results and nitrogen cycling genes for metagenome assembled genomes from Jewel Lake enrichment
