# Supplementary figures and images for: Selective carbon sources influence the end products of microbial nitrate respiration
Source: ISME J. 2020 May 5;14(8):2034–45. doi: 10.1038/s41396-020-0666-7 (PMC7368043; doi:10.1038/s41396-020-0666-7)

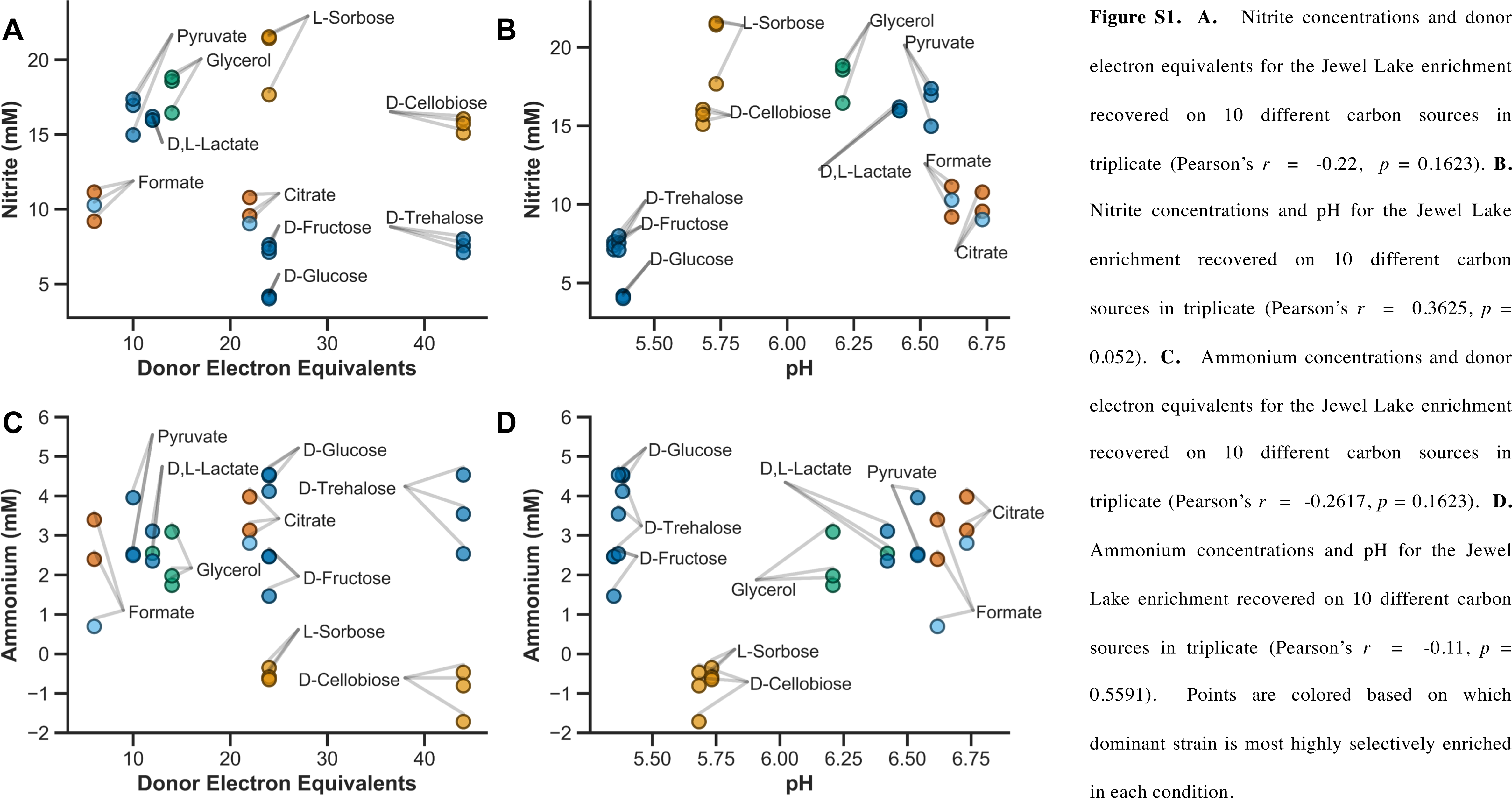

Supplement: Supplementary file 2 — Figure S1. [file 41396_2020_666_MOESM2_ESM.tif]

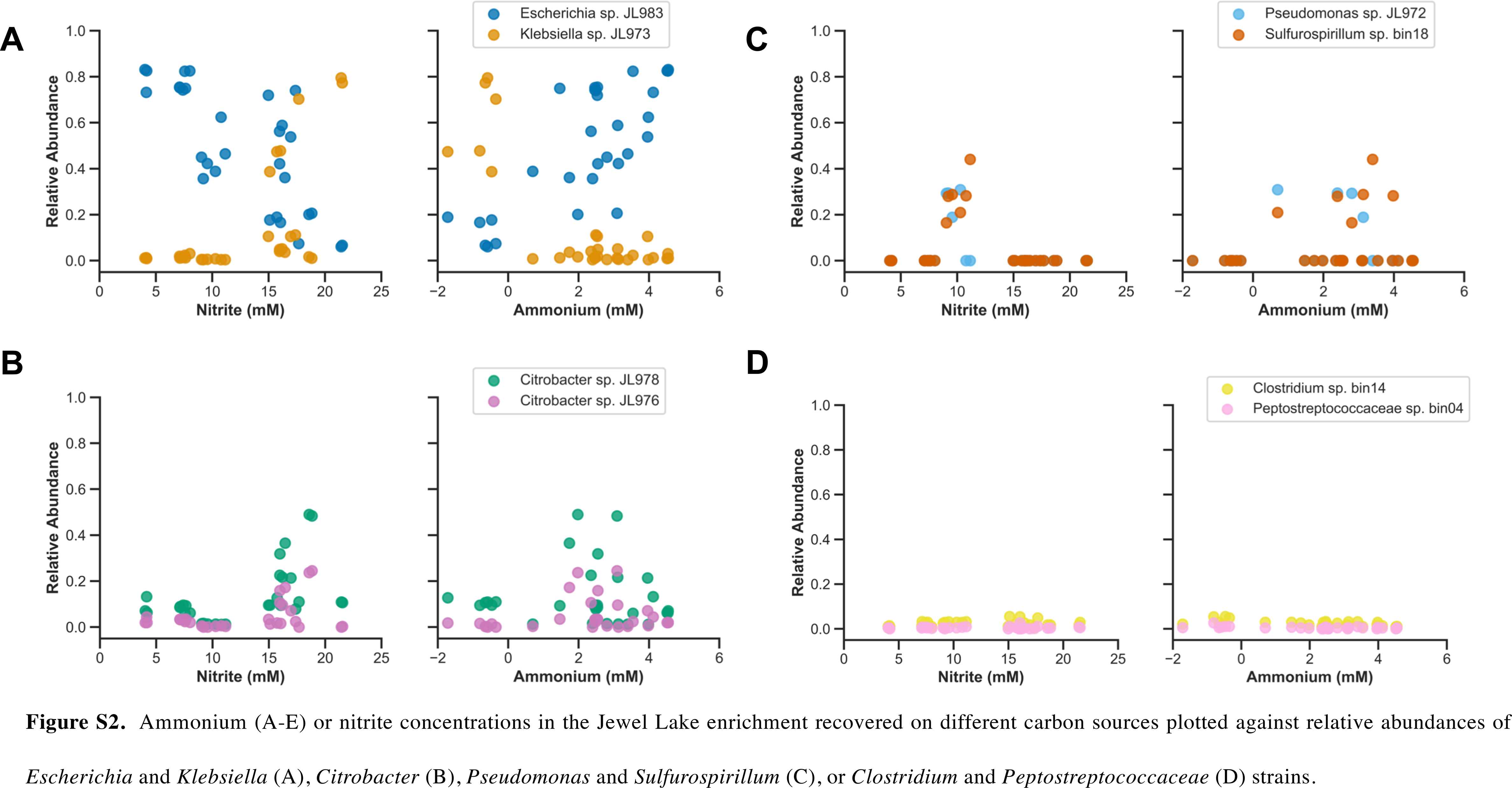

Supplement: Supplementary file 3 — Figure S2. [file 41396_2020_666_MOESM3_ESM.tif]
